# Confirmation for conformational selection

**DOI:** 10.7554/eLife.34923

**Published:** 2018-02-20

**Authors:** Yajun Jiang, Charalampos G Kalodimos

**Affiliations:** Department of Structural BiologySt Jude Children's Research HospitalMemphisUnited States

**Keywords:** Hsp70, conformational selection, induced fit, molecular chaperones, methyl-TROSY NMR, *E. coli*

## Abstract

NMR studies settle part of a long-standing debate about the mechanism used by the Hsp70 chaperone to recognize substrates.

**Related research article** Sekhar A, Velyvis A, Zoltsman G, Rosenzweig R, Bouvignies G, Kay L. 2018. Conserved conformational selection mechanism of Hsp70 chaperone-substrate interactions. *eLife* **7**:e32764. doi: 10.7554/eLife.e32764

Almost every process in biology relies on proteins in one way or another, and most of these proteins need to have a specific three-dimensional structure to carry out their roles. A network of molecular machines – called chaperones – makes sure that proteins tend to end up folded correctly (Balchin et al., 2016), and a chaperone called Hsp70 is a central hub in this network.

Hsp70 has a domain that binds to substrates at one end (Mayer and Bukau, 2005), and a domain that binds to nucleotides like ATP at the other end. The chaperone’s affinity for substrates decreases when ATP binds to this domain, and it increases again when the ATP is broken down by hydrolysis to yield ADP. Importantly, Hsp70 does not work alone; another protein called Hsp40 both helps to deliver substrates to Hsp70 and also catalyzes the hydrolysis of ATP.

However, despite much progress in recent years (Clerico et al., 2015; Mayer and Kityk, 2015), major questions remain about the interactions between Hsp70 and its substrates. For instance, does Hsp70 passively bind to exposed segments of unfolded proteins, as proposed in the ‘conformational selection’ hypothesis, or does it actively unfold misfolded substrates, as proposed by the ‘induced fit’ hypothesis? Now, in eLife, Ashok Sekhar, Lewis Kay and colleagues report an answer to this long-standing question (Sekhar et al., 2018).

It has proven challenging to decide between these two hypotheses, partly because chaperones are highly dynamic molecular machines, which limits the number of techniques that can be used to study them in action. Fortunately, NMR spectroscopy provided a perfect solution to tackle this problem (Kay, 2016; Huang and Kalodimos, 2017).

According to the conformational selection hypothesis, the substrates switch between being folded and unfolded, and Hsp70 selectively binds to the unfolded state (Figure 1A). The induced fit hypothesis, however, proposes that Hsp70 binds to a folded or misfolded substrate and then unfolds it (Figure 1B). Therefore, if someone could directly detect a substrate switching between an unfolded state and an Hsp70 bound state, that would be evidence for the conformational selection hypothesis. No such exchange should occur if the induced fit model is correct.

**Figure 1.**
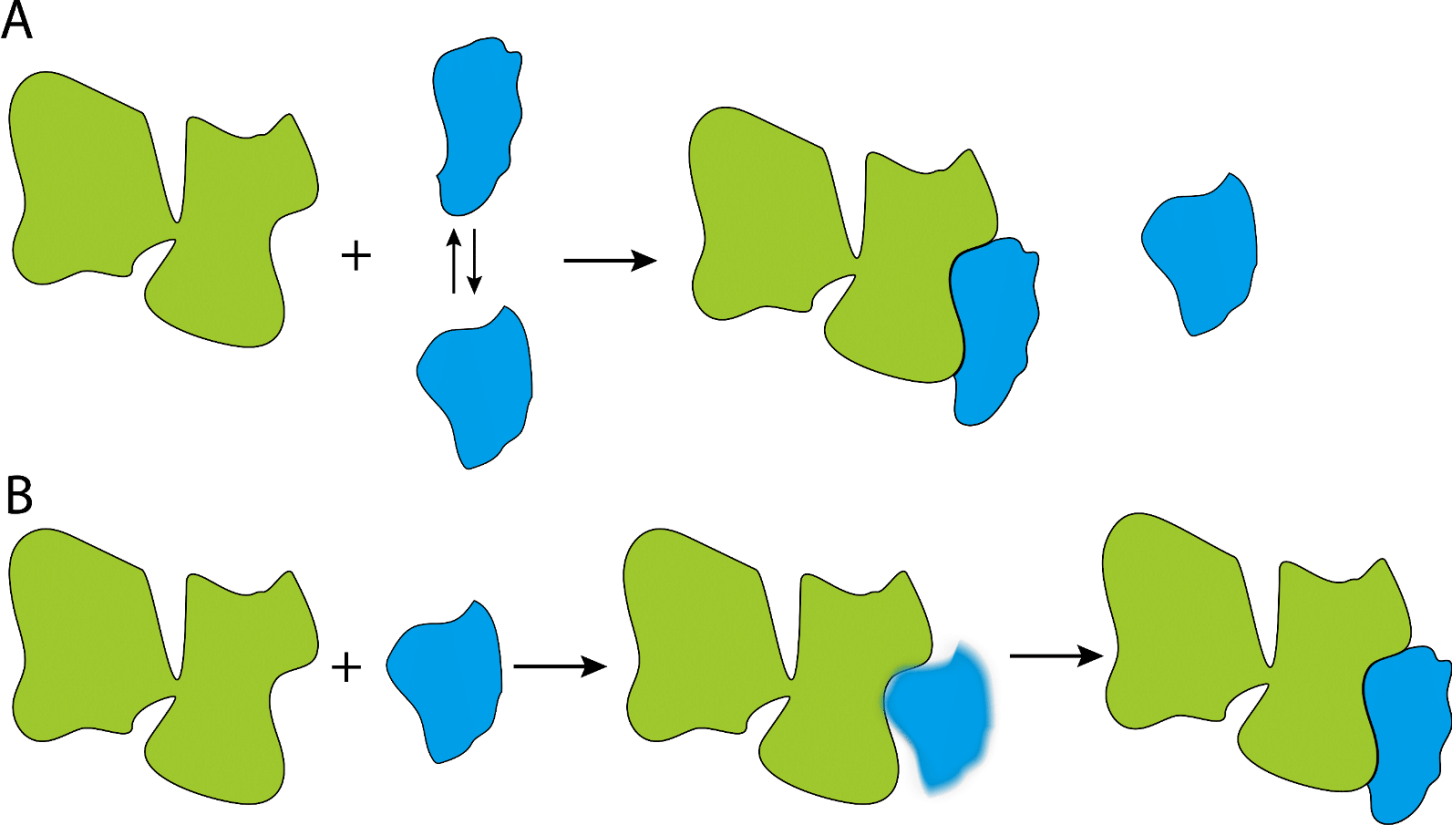
Cartoon illustrations of the conformational selection and induced fit mechanisms. (**A**) In the conformational selection mechanism, the substrate (blue) switches between different conformations, and the chaperone (green) selectively interacts with one of these conformations. (**B**). In the induced fit mechanism, the chaperone directly interacts with the substrate, irrespective of the latter's conformation, and then changes its conformation.

Using advanced NMR methodologies, Sekhar et al. – who are based at the University of Toronto, the Weizmann Institute of Science, the École Normale Supérieure in Paris and the Hospital for Sick Children, also in Toronto – could keep track of two processes – magnetization exchange and chemical exchange – as Hsp70 interacted with a model substrate. Roughly speaking, these two processes can be used to measure conformational changes undergone by the substrate during its interaction with Hsp70.

Sekhar et al. found that Hsp70 from humans and a related bacterial chaperone called DnaK both interact with substrates through the conformational selection mechanism. It was already known that DnaK behaves in a similar way to human Hsp70 (Clerico et al., 2015; Mayer and Kityk, 2015). The results of Sekhar et al. suggest, therefore, that the Hsp70 chaperone machinery prevents the misfolding of proteins by selectively binding to unfolded substrates instead of actively unfolding substrates that have misfolded. The methods reported by Sekhar et al. could also be used to study other chaperone systems such as the Trigger Factor (Saio et al., 2014) and SecB (Huang et al., 2016), both of which capture their substrate proteins in an unfolded state.

In vivo, the Hsp70 machinery needs to process long proteins consisting of more than 300 to 400 amino acids (Balchin et al., 2016). Yet Hsp70 has just one relatively small substrate-binding site that can accommodate only seven to eight amino acids. How can Hsp70 fulfill such a challenging task? It is thought that Hsp40 helps deliver substrates to Hsp70, and so it will be important to examine the exact role that Hsp40 plays in recognizing substrates and delivering them to Hsp70. Will the current model, which works for isolated Hsp70, still apply when Hsp40 and the rest of the Hsp70 machinery are present? Many key questions remain unanswered, yet this latest study gives hope that NMR spectroscopy is well suited to address these questions.

## References

[bib1] Balchin D, Hayer-Hartl M, Hartl FU (2016). In vivo aspects of protein folding and quality control. Science.

[bib2] Clerico EM, Tilitsky JM, Meng W, Gierasch LM (2015). How Hsp70 molecular machines interact with their substrates to mediate diverse physiological functions. Journal of Molecular Biology.

[bib3] Huang C, Rossi P, Saio T, Kalodimos CG (2016). Structural basis for the antifolding activity of a molecular chaperone. Nature.

[bib4] Huang C, Kalodimos CG (2017). Structures of large protein complexes determined by nuclear magnetic resonance spectroscopy. Annual Review of Biophysics.

[bib5] Kay LE (2016). New views of functionally dynamic proteins by solution NMR spectroscopy. Journal of Molecular Biology.

[bib6] Mayer MP, Bukau B (2005). Hsp70 chaperones: cellular functions and molecular mechanism. Cellular and Molecular Life Sciences.

[bib7] Mayer MP, Kityk R (2015). Insights into the molecular mechanism of allostery in Hsp70s. Frontiers in Molecular Biosciences.

[bib8] Saio T, Guan X, Rossi P, Economou A, Kalodimos CG (2014). Structural basis for protein antiaggregation activity of the trigger factor chaperone. Science.

[bib9] Sekhar A, Velyvis A, Zoltsman G, Rosenzweig R, Bouvignies G, Kay L (2018). Conserved conformational selection mechanism of Hsp70 chaperone-substrate interactions. eLife.

